# Impact of intensified contact precautions while treating hematopoietic stem cell transplantation recipients during aplasia

**DOI:** 10.1186/s40001-023-01085-8

**Published:** 2023-03-15

**Authors:** Christian Böing, Christian Reicherts, Neele Froböse, Alexander Mellmann, Frieder Schaumburg, Georg Lenz, Stefanie Kampmeier, Matthias Stelljes

**Affiliations:** 1 Institute of Hygiene, University Hospital Münster, Robert-Koch-Straße 41, 48149 Münster, Germany; 2 Department of Medicine A, Hematology and Oncology, University Hospital Münster, Albert-Schweitzer-Campus 1, 48149 Münster, Germany; 3 Institute of Medical Microbiology, University Hospital Münster, Domagkstraße 10, 48149 Münster, Germany

**Keywords:** HCT recipients, Hospital-acquired bloodstream infections, Infection control bundle strategies

## Abstract

**Background:**

Bacterial infections are a major complication for patients undergoing allogeneic hematopoietic stem cell transplantation (HCT). Therefore, protective isolation is considered crucial to prevent nosocomial infections in this population. Here, the impact of intensified contact precautions on environmental contamination and the occurrence of bloodstream infections (BSI) in patients on a HCT unit were compared between two contact precaution measures.

**Methods:**

A 2-year retrospective observational study was performed. In the first year, strict contact precaution measures were applied (i.e., protective isolation, the use of sterile personal protective equipment (PPE) by healthcare workers and visitors and sterilization of linen and objects that entered the patient’s room). After one year, contact precautions were reduced (i.e., no use of sterile PPE, no sterilization of linen and objects that entered the patient’s room). Environmental contamination in randomly selected patient rooms was monitored by sampling six standardized environmental sites in the respective patient treatment units. In a before-and-after study, the number of BSI episodes of those patients, who were accommodated in the monitored rooms was compared.

**Results:**

In total, 181 treatment units were monitored. No significant difference in the contamination of anterooms and patient’s rooms between both groups was found. A total of 168 patients were followed for the occurrence of BSI during the entire study period (before: 84 patients, after: 84 patients). The total count of patients with BSI episodes showed a higher incidence in the period with reduced contact precautions (30/84 vs. 17/84, *p* = 0.039). The cause of this increasing number of BSI can be traced back to BSI episodes with common commensal bacteria (17/84 vs. 5/84, *p* = 0.011).

**Conclusions:**

The implementation of maximal barrier measures did not reduce the bacterial contamination of the patients’ environment. The impact on the patients’ outcomes remain controversial. Further research is needed to investigate the impact of infection prevention measures on the clinical outcome of patients undergoing HCT.

## Introduction

Patients undergoing hematopoietic stem cell transplantation (HCT) are susceptible to infections due to a compromised immune system. Infections in these aplastic patients remain a relevant cause for mortality and morbidity [1]. In this context, bloodstream infections (BSI) pose a major threat in HCT recipients occurring in 20 to 45% of all HCT recipients [2–8].

Maximal barrier measures are used to prevent nosocomial infections in those vulnerable patients, but the evidence for certain recommendations is limited. Consequently, maximal barrier measures are implemented inconsistently across centers [9–13]. Since the beginning of high-dose chemotherapy and HCT in the 1960s, strict preventive isolation measures have been introduced in neutropenic patients [9], often as part of extensive infection control bundle strategies including physical barriers (gloves, gowns, etc.), hand hygiene, antibiotic regimens, low-bacterial or sterile food and air-filtering technologies [10, 11, 14]. However, extensive hygiene measures are cost intensive, time-consuming and have negative effects on patient’s health [10, 15, 16]. Hence, especially maximal contact precautions have been evaluated in recent decades, while the benefit of strict isolation was questioned [9, 17–19]. Currently, we are experiencing a shift in protective isolation practice and more hygienic measures of protective isolation are abandoned [9]. Here, we investigated the role of strict vs. reduced infection control barrier measures on the prevention of nosocomial infections, i.e., life-threatening BSI.

## Methods

### Patients and treatment

The Bone Marrow Transplantation unit of the University Hospital Muenster serves a catchment area of 5 million people in the northwest of Germany. It consists of an outpatient clinic and an interdisciplinary inpatient unit with 20 high-efficiency particulate air (HEPA)–filtered single-patient rooms, all of which were equipped with separate bathrooms and anterooms.

During the study period (January 2018–December 2019), 320 adult patients underwent HCT, accounting for a total of 361 admissions, including 41 readmissions for transplant-associated complications. Key demographic and medical characteristics of the patients are summarized in Table 1. In general, patients undergoing allogeneic HCT are treated for 4–6 weeks as inpatients. Standard prophylactic antibiotic treatment, starting with initiation of conditioning regimen, consisted basically of ciprofloxacin or, in the case of allergy, intolerance or concurrent medical conditions, amoxicillin, respectively. Standard empiric antibacterial therapy for fever of unknown origin was piperacillin/tazobactam, combined with an aminoglycoside (AG) in case of fever onset in neutropenia. AG was substituted by tigecycline in case of severe sepsis or septic shock, preexisting neutropenia since months before conditioning. BSI are treated according to guidelines of the European Society of Clinical Microbiology and Infectious Diseases (ESCMID).

**Table 1:** Characteristics of patients treated at the HCT unit

Characteristic	2018	2019
No. of pat. for allogeneic transplantation	162	158
Age		
Median (range), years	58 (19–75)	59 (19–74)
Sex		
Male, *n* (%)	106 (65)	90 (57)
Female, *n* (%)	56 (35)	68 (43)
Conditioning regimen		
Myeloablative conditioning, *n* (%)	59 (36)	60 (38)
Reduced intensity conditioning, *n* (%)	71 (44)	68 (43)
Sequential conditioning, *n* (%)	32 (20)	30 (19)
Status at start of cond		
Complete remission, *n* (%)	66 (41)	60 (38)
Active disease, *n* (%)	96 (59)	98 (62)
Diseases		
Acute myeloid leukemia, n (%)	85 (53)	80 (51)
Acute lymphoblastic leukemia, n (%)	15 (9)	12 (8)
Myelodysplastic syndrome, *n* (%)	24 (15)	18 (11)
Myeloproliferative neoplasia, *n* (%)	12 (7)	22 (14)
Lymphoma, *n* (%)	24 (15)	19 (12)
Multiple myeloma, *n* (%)	2 (1)	5 (3)
Aplastic anemia, *n* (%)	0 (0)	2 (1)
Duration of neutropenia after transplant		
Median (range), days	16 (4–56)	16 (0–56)
Duration of hospitalization after transplant		
Median (range), days	30 (10–155)	30 (6–178)
Patients with readmissions after transplant		
Total, *n*	23	18
Infections, *n*	7	1
Graft-versus-host disease, *n*	12	9
Other, *n*	4^a^	8^b^

^a^unspecific diarrhea, subileus, acute encephalopathy, portal hypertension

^b^CAR-T-call therapy, blinatumomab therapy, relapse

### Study setting and design

We conducted a retrospective study in our center in 2018 (January 1st–December 31st) and 2019 (January 1st–December 31st), during which our in-house standard on hygiene measures for the BMT unit was changed. As part of these changes, the hygiene measures have been reduced in 2019 compared to 2018 as described below. Data are presented as anonymized, cumulative information. All epidemiological surveillance strategies and investigations were performed in accordance with the national recommendations for treatment of immunocompromised patients of the German legally assigned institute for infection control and prevention [20].

### Hygienic measures

During the control period (2018), a bundle of strict measures for protective isolation has been implemented. Medical staff and visitors, before entering the BMT unit, dressed in functional clothing and performed hygienic hand disinfection. Medical staff and visitors wore sterile gloves and gowns in addition to caps and masks when entering the patient’s room. All objects (linen, jars, cups, newspapers, toilet paper, etc.) entered the patient’s room sterilized by autoclaving. Disinfection of surfaces was performed once a day. During the following period (2019), a bundle of hygienic measures was abandoned. The use of sterile gloves and gowns was ceased and instead low-germ gowns and standard hand hygiene were implemented. Sterile gloves were still used when aseptic tasks or manipulation at central venous lines were performed. Autoclaving of linen and objects that entered the patient’s room was quitted.

### Environmental monitoring

Hygienic monitoring in rooms of aplastic patients was conducted via swab sampling (Transwab^®^ m40 compliant, mwe, Corsham, Wiltshire, UK). Hygiene monitoring was carried out regularly throughout the entire study period. Monitoring of one to three rooms was carried out weekly or every second week. All rooms examined were single rooms. We monitored surfaces of six different environmental sites including two in the patient’s room (chair and table; bedside table) and three in the anteroom (infusion pumps; handles of the cabinets; computer, keyboard and mouse). Additionally, the handles of the door separating anteroom and patient’s room were sampled. Swabs were streaked on Colombia sheep (Oxoid, Wesel, Germany) blood agar and incubated for 48 h at ambient air. Species were identified by matrix-assisted laser desorption/ionization-time of flight-mass spectrometry (MALDI-TOF MS; Bruker, Bremen, Germany) using the biotyper database (Bruker). The identified bacterial species during environmental monitoring were assigned to the group of obligatory pathogenic bacteria or skin and environmental bacteria. This classification was based on the following hygienic considerations. Contaminations with skin and environment bacteria of surfaces in the hospital have a different significance from a hygienic point of view compared to obligatory pathogenic bacteria. This is due to their higher pathogenicity, presence of antibiotic resistance and their patient-to-patient transmission potential. Hence, *Escherichia coli*, *Klebsiella pneumoniae*, *Enterococcus faecium*, *Enterococcus faecalis* and *Staphylococcus aureus* were defined as obligatory pathogenic bacteria, while coagulase-negative staphylococci (CNS), oral streptococci and *Bacillus* spp. were defined as skin or environmental bacteria (see also Table 2).

**Table 2:** Obligatory pathogenic and skin/environmental bacteria in the patient’s environment

Species	2018 *n* = 89	2019 *n* = 92
Obligatory pathogenic bacteria		
*Staphylococcus aureus*	10 (11.2%)	10 (10.9%)
*Enterococcus faecium/faecalis*	13 (14.6%)	15 (16.3%)
GNB^a^	1 (1.1%)	3 (3.3%)
Skin and environmental bacteria		
CNS^b^	66 (74.2%)	71 (77.2%)
*Bacillus* spp.	42 (47.2%)	26 (28.3%)
viridans *Streptococci*	15 (16.9%)	4 (4.3%)
*Acinetobacter* spp.^c^	2 (2.2%)	0
*Staphylococcus pseudintermedius*	1 (1.1%)	0
*Corynebacterium* ssp.	1 (1.1%)	0

^a^GNB; Gram-negative bacteria

^b^CNS; coagulase-negative *Staphylococci*

^c^No *Acinetobacter baumannii* complex

### Blood cultures sampling and BSI

Blood cultures were taken from patients who were lying in a patient room at the time of the environmental monitoring and who developed fever during any time of transplant hospitalization. BSI was defined as an episode with typical clinical characteristics where the patient had to have at least one of the following signs: fever (> 38 °C, chills or hypotension) [21] and, depending on detected microorganisms, one positive blood sample in case of obligatory pathogenic bacteria or separate positive blood samples with identical species in at least two separate blood cultures in case of common commensal bacteria (see NHSN Organisms List [21]).

An automated blood culture system (BACTECT 9240, BD, Heidelberg, Germany) was used to detect positive blood culture samples. These were plated on Colombia blood agar. Species identification was performed using MALDI-TOF MS. Retrospectively, cultivated species from blood cultures and pathogens derived from environmental sampling sites of the patient’s room were compared.

### Statistical analysis

Descriptive statistic was expressed by total number and percentage for categorical variables. Univariate analysis of categorical variables was performed using the Chi-squared test and Fisher’s exact test. A *p*-value $<$ 0.05 was considered statistically significant. All statistical analyses were performed using R Studio version 2022.07.0 + 548 (R version 4.2.1) (The R Foundation, Vienna, Austria).

## Results

### Patients’ characteristics

In 2018 and 2019, 162 and 158 patients, respectively, underwent an allogeneic HCT. Patients’ characteristics in both years were well balanced with regard to age, sex, duration of severe neutropenia, intensity of conditioning therapy, underlying malignant disease, remission status prior transplantation conditioning therapies and duration of hospitalization (Table 1).

### Environmental contamination

In total, 181 patients’ rooms with adjoining anterooms were monitored during the 2-year study period (2018: 89 rooms, 2019: 92 rooms). The contaminations were mainly caused by Gram-positive bacteria, whereas Gram-negative bacteria were rarely found on the surfaces. In 2018, 10 (11.2%) patient rooms showed lower contamination with obligatory pathogenic bacteria compared to 17 (18.5%) rooms in 2019. The total contamination with obligatory pathogenic bacteria of the anteroom decreased from 12 (13.5%) in 2018 to 10 (10.9%) in 2019. No significant difference of contamination of patients’ rooms and anterooms with obligatory pathogenic bacteria could be identified between both study groups (*p* = 0.247 and *p* = 0.756, respectively)**.** The extent of contamination of skin and environmental bacteria in the patient rooms (2018: *n* = 48 (53.9%), 2019: *n* = 49 (53.3%)) and anterooms (2018: *n* = 71 (79.8%), 2019: *n* = 69 (75.0%)) was not statistical different between both study groups (*p* = 1 and 0.555, respectively). *S. aureus* and *E. faecium/E. faecalis* were the most frequent obligatory pathogenic bacteria in the patients’ environments (Table 2). Most contaminated locations with obligatory pathogenic bacteria were surfaces of the chair and table, the bedside table, infusion pumps and the computer, keyboard and mouse (Table 3).

**Table 3:** Location of contamination with obligatory pathogenic bacteria and skin/environmental bacteria

Locations	Obligatory pathogenic bacteria			Skin and environmental bacteria		
	2018	2019	*p*-value	2018	2019	*p*-value
Patient’s room						
Chair and table	7 (7.9%)	12 (13.0%)	0.371	36 (40.4%)	38 (41.3%)	1
Bedside table	6 (6.7%)	5 (5.4%)	0.955	32 (35.9%)	43 (46.7%)	0.186
Anteroom						
Handles of door	1 (1.1%)	0 (0%)	–	8 (9.0%)	13 (14.1%)	0.397
Computer, keyboard and mouse	10 (11.2%)	6 (6.5%)	0.393	61 (65.5%)	49 (53.3%)	0.051
Infusion pumps	4 (4.5%)	6 (6.5%)	0.747	39 (43.8%)	34 (37.0%)	0.430
Handles of the cabinets	0 (0%)	0 (0%)	–	17 (19.1%)	12 (13.0%)	0.364

### Bloodstream infections

A total of 168 patients were screened for BSI. In each year, 84 patients were followed. The most common bacteria were *S. epidermidis* (*n* = 14 (8.5%)), *E. coli* (*n* = 13 (7.9%)), viridans *Streptococci* (*n* = 11 (6.7%)) and *E. faecalis/E. faecium* (*n* = 8 (4.8%), Table 4). In 2019, 30 episodes of BSI occurred compared with 17 periods in 2018, *p* = 0.039. Interestingly, this increase of BSI was caused by the increase of BSI with common commensal bacteria and less by bacteria classified as obligatory pathogenic bacteria (2018: *n* = 5 (6.0%), 2019: *n* = 17 (20.2%), *p* = 0.011). The prevalence of BSI caused by obligatory pathogenic bacteria was relatively stable with 12 (14.3%) BSI and 13 (15.5%) BSI in 2018 and 2019, respectively, *p* = 1.

**Table 4:** Bacteria isolated from blood cultures

Species^a^	2018	2019
*Enterococcus faecium/faecalis*	3	5
*Klebsiella pneumoniae*	2	0
*Escherichia coli*	6	7
*Staphylococcus aureus*	1	0
*Pseudomonas aeruginosa*	0	1
*Stenotrophomonas maltophilia*	1	0
*Candida glabrata*	1	0
CNS^b^	6	8
viridans *Streptococci*	4	7
*Rothia mucilaginosa*	0	5
*Leptotrichia trevisanii*	0	1
*Fusobacterium nucleatum*	0	1
*Actinomyces odontolyticus*	1	1

^a^All pathogens that are considered causative for the development of a bloodstream infection are listed. In some cases, multiple pathogens have been considered causative in the context of a bloodstream infection, therefore the sum of pathogens detected is greater than the number of bloodstream infections detected

^b^CNS: coagulase-negative* Staphylococci*

## Discussion

Our study revealed that reduced infection control barrier measures during protective isolation did not increase contamination of the patients’ surroundings with obligatory pathogenic or skin and environmental bacteria in our HCT center. These results suggest that intensified contact precautions including sterile gloves and gowns as well as sterilization of linen and all objects that enter the patient rooms is not necessary in reducing bacterial contamination of the patients’ environment, if standard hygienic bundle strategies are routinely implemented. Especially, the contamination of surfaces in the anteroom by obligatory pathogenic bacteria was low. The prevalence of BSI caused by obligatory pathogenic bacteria remained stable after control barrier measures were reduced. Interestingly, in the group with reduced measures, an increase of BSI with common commensal bacteria was found. Zeneli et al*.* found no correlation of environmental microbial load and infections in HCT recipients [22]. The transmission of obligatory pathogenic bacteria from the environment to the patients resulting in severe infections seems to be rather limited even in patients with hematopoietic stem cell transplantation. Because of the missing correlation of bacterial contamination of the environment and the prevalence of BSI, endogenous infections is considered to be the predominant etiology of infections in patients undergoing HCT as published previously [11, 23]. Clinical strategies to prevent infections in patients undergoing HCT should especially focus on prevention of endogenous infections with the help of standard hygienic measures as hand disinfection and surface disinfection. In addition, strict hygiene measures should be implemented especially when working aseptically. In this context, the benefit of extended hygiene measures, such as those described here, must be questioned. However, considering the COVID-19 pandemic, future research should investigate the effect of extended measures on other infections, such as respiratory or *Clostridioides difficile* infections (CDI).

There are some limitations of our study. First, we did not monitor the patient’s environment explicitly at the moment when BSI were present, but rather in an interval at an average of around three to four weeks. Therefore, a transmission from the environment to the patient or vice versa cannot be ruled out. Second, we did not quantify the amount of bacterial load of contaminations on the surfaces. Samples were collected by using swabs and therefore only a qualitative evaluation of bacterial colonies was performed. This, on the other hand, is of minor importance because the detection of obligatory pathogenic bacteria in the environment of HCT recipients is considered a relevant contamination irrespective of the quantity of the bacterial load. Third, before-and-after studies, i.e., interrupted time series are themselves susceptible to various biases [24]. Potential biases of our study are history bias (e.g., different strategies for sampling and antimicrobial prophylaxes) and selection bias.

## Conclusion

The clinical impact of maximal infection control barrier measures for high-risk patients remains controversial. Standardized hygienic bundle strategies should focus on contact precautions, disinfection strategies and antimicrobial stewardship initiatives. As a consequence of our study, we have continued environmental sampling for further surveillance and implemented short teaching sessions.

## Data Availability

The datasets used and/or analyzed during the current study are available from the corresponding author on reasonable request.

## Abbreviations

BMT: Bone marrow transplant
BSI: Bloodstream infections
HEPA: High-efficiency particulate air
HCT: Hematopoietic stem cell transplantation
PPE: Personal protective equipment
